# Brain-Derived Neurotrophic Factor (BDNF) Preserves the Functional Integrity of Neural Networks in the β-Amyloidopathy Model *in vitro*

**DOI:** 10.3389/fcell.2020.00582

**Published:** 2020-07-08

**Authors:** Elena V. Mitroshina, Roman S. Yarkov, Tatiana A. Mishchenko, Victoria G. Krut’, Maria S. Gavrish, Ekaterina A. Epifanova, Alexey A. Babaev, Maria V. Vedunova

**Affiliations:** ^1^Department of Neurotechnology, Institute of Biology and Biomedicine, National Research Lobachevsky State University of Nizhny Novgorod, Nizhny Novgorod, Russia; ^2^Molecular and Cell Technologies Group, Central Scientific Research Laboratory, Privolzhsky Research Medical University, Nizhny Novgorod, Russia

**Keywords:** neural networks, Alzheimer’s disease, β-amyloidopathy, brain-derived neurotrophic factor, microelectrode arrays, calcium imaging, neuroprotection

## Abstract

Alzheimer’s disease (AD) is a widespread chronic neurodegenerative pathology characterized by synaptic dysfunction, partial neuronal death, cognitive decline and memory impairments. The major hallmarks of AD are extracellular senile amyloid plaques formed by various types of amyloid proteins (Aβ) and the formation and accumulation of intracellular neurofibrillary tangles. However, there is a lack of relevant experimental models for studying changes in neural network activity, the features of intercellular signaling or the effects of drugs on the functional activity of nervous cells during AD development. In this work, we examined two experimental models of amyloidopathy using primary hippocampal cultures. The first model involves the embryonic brains of 5xFAD mice; the second uses chronic application of amyloid beta 1-42 (Aβ1-42). The model based on primary hippocampal cells obtained from 5xFAD mice demonstrated changes in spontaneous network calcium activity characterized by a decrease in the number of cells exhibiting Ca^2+^ activity, a decrease in the number of Ca^2+^ oscillations and an increase in the duration of Ca^2+^ events from day 21 of culture development *in vitro*. Chronic application of Aβ1-42 resulted in the rapid establishment of significant neurodegenerative changes in primary hippocampal cultures, leading to marked impairments in neural network calcium activity and increased cell death. Using this model and multielectrode arrays, we studied the influence of amyloidopathy on spontaneous bioelectrical neural network activity in primary hippocampal cultures. It was shown that chronic Aβ application decreased the number of network bursts and spikes in a burst. The spatial structure of neural networks was also disturbed that characterized by reduction in both the number of key network elements (hubs) and connections between network elements. Moreover, application of brain-derived neurotrophic factor (BDNF) recombinant protein and BDNF hyperexpression by an adeno-associated virus vector partially prevented these amyloidopathy-induced neurodegenerative phenomena. BDNF maintained cell viability and spontaneous bioelectrical and calcium network activity in primary hippocampal cultures.

## Introduction

Alzheimer’s disease (AD) studies are becoming more relevant each year due to the increase in the life expectancy of the population and the accumulation of information regarding AD polyetiology (Dubois et al., 2014; Hampel et al., 2015). The features of AD pathological processes and the development of new strategies to prevent neurodegeneration are actively pursued worldwide (Hadar and Gurwitz, 2018; Cao et al., 2018; Wang N. et al., 2018). Nevertheless, there is no clear therapeutic solution for highly accelerated neurodegeneration, even if it is diagnosed at an early stage.

Investigations of AD processes have raised questions about the possibility of using endogenous regulatory molecules, such as neurotrophic factors, to correct neurodegeneration at different stages of pathology development. The content of neurotrophic factors decreases with neurodegeneration, and this process correlates with AD stages (Peng et al., 2005; Wang and Holsinger, 2018). Brain-derived neurotrophic factor (BDNF) is a potent biological agent that maintains cell viability and functional neuron activity in various pathological states, including severe genetically determined neurodegenerative diseases (Criscuolo et al., 2015; de Pins et al., 2019; Choi et al., 2018). Viral constructs carrying the BDNF gene are a promising therapeutic strategy to restore BDNF levels in the brain. Several studies have indicated the efficacy of viral vectors carrying neurotrophic factor genes in the treatment of Parkinson’s disease (Lim et al., 2010; Cheng et al., 2018; Tereshchenko et al., 2014). The establishment of approaches to use viral constructs carrying the BDNF gene in AD has been carrying out for the past 10 years (Nagahara et al., 2009; Nagahara et al., 2013; Jiao et al., 2016).

AD is characterized by significant variability in the age of disease manifestation and the rate of disease progression. The major hallmarks of AD are the pathological accumulation of amyloid beta (Aβ) protein in the form of extracellular plaques in brain parenchyma and capillaries and the abnormal phosphorylation of tau protein, which forms neurofibrillary tangles (Bourdenx et al., 2017). Aggregation of Aβ and phosphorylated tau occurs gradually; monomers are aggregated into oligomers in neurons and then collected into fibrils, leading to the formation of amyloid plaques and neurofibrillary tangles (Walker et al., 2013; Cline et al., 2018).

The elaboration of experimental models of AD is a key for better understanding AD pathogenesis and assessing the potential of new therapeutic approaches for effective neurodegenerative process correction (Drummond and Wisniewski, 2017). *In vivo* models are currently the most frequently used experimental models of AD and are mostly based on transgenic mice that overexpress human genes associated with the familial form of AD (such as the familial AD (FAD) lines), resulting in the formation of amyloid plaques (Mucke et al., 2000; Webster et al., 2014).

The adequacy of any biological or mathematical model depends on specific tasks and possible approaches to its solution. Investigation of neural networks as the minimal functional unit of the nervous system responsible for the processes of reconsolidation and storage of information is considered one of the principal aspects of studies on the neurodegeneration processes. A neural network is not only a functionally connected complex of neurons but also a single functional ensemble capable of responding in a consolidated manner to changes caused by both external and internal stimuli (Yuste, 2015; Mishchenko et al., 2019). A single neural network in the native brain is extremely difficult to study and cannot be examined in a comprehensive manner at this time. Primary hippocampal cultures are considered an adequate biological model that allows the study of individual cellular and network reactions under stress and the effects of neuroprotectants in a chronic experiment with the possibility of multiple measurements of neural network activity (Johnstone et al., 2010; Vedunova et al., 2015; Hasan and Berdichevsky, 2016). To investigate the changes in functional neural network activity caused by AD development, we used a protocol for creating primary neuronal cultures obtained from 5xFAD murine embryos. Notably, however, models using transgenic animals mostly simulate familial forms of AD, which account for only 5% of all cases of this pathology (Bilkei-Gorzo, 2014; Karch et al., 2014; Kim et al., 2014). These models often lack a complex of pathological traits exhibited by patients with AD. These transgenic mice are characterized by amyloid plaques, compromised synaptic transmission and memory impairment, but these symptoms are not always accompanied by neuronal loss and, most importantly, neurofibrillary tangle formation (Oakley et al., 2006). The poor correlation between preclinical research of new therapeutic drugs and clinical trials is probably associated with this issue (Banik et al., 2015; Cummings et al., 2018). Therefore, the development of relevant experimental models will provide a more complete view of pathogenic processes in AD. Such opportunities may be possible in an *in vitro* amyloidosis model based on synthetic amyloid peptide application (Stancu et al., 2014; Villalobos Acosta et al., 2018; Mango et al., 2019).

Our present study is devoted to adapting an *in vitro* amyloidopathy model that allows investigation of the functional activity of neural networks. Using our model, we also studied the influence of BDNF on cell viability and the reorganization of neural networks in AD development.

## Materials and Methods

### Ethics Statement

All experimental protocols used in this study were approved by the Bioethics Committee of Lobachevsky University and carried out in accordance with Act708n (23.08.2010) of the Russian Federation National Ministry of Public Health, which states the rules of laboratory practice for the care and use of laboratory animals, and the Council Directive 2010/63 EU of the European Parliament (September 22, 2010) on the protection of animals used for scientific purposes. C57BL/6J mice were killed by cervical vertebra dislocation, and their embryos were then surgically removed and sacrificed by decapitation.

### Primary Neuronal Cultures

Primary hippocampal cells were obtained from murine embryos (day 18 of gestation). A detailed protocol for culture preparation is described in Vedunova et al., 2015. Hippocampi were surgically isolated. Cell dissociation was achieved through mechanical dissection followed by incubation for 20 min in 0.25% trypsin-EDTA solution (Gibco, 25200056, United States). The obtained cell suspension was centrifuged at 1000 rpm for 3 min. Then, the cell pellet was resuspended in Neurobasal^TM^ medium (Gibco, 21103049, United States) supplemented with 2% B27 (Gibco, 175040446, United States), 0.5 mM L-glutamine (Gibco, 25030024, United States) and 5% fetal bovine serum (FBS) (PanEco, K055, Russia). To perform a viability assessment, immunocytochemical analysis and registration of functional calcium activity, we placed cells on coverslips (18x18 mm) pretreated with polyethyleneimine solution (1 mg/mL) (Sigma-Aldrich, Germany). For electrophysiological experiments, cells were cultured on multielectrode arrays (MEAs; MEA60, Multichannel, Germany). The initial density of cells was 9000 cells/mm^2^. Half of the medium containing 0.4% FBS was replaced every third day. Cell viability was maintained under constant conditions of 35.5°C, 5% CO_2_ and a humidified atmosphere in a CO_2_ incubator (Sheldon Manufacturing, United States).

### 5xFAD Embryo Genotyping

To obtain primary hippocampal cultures from 5xFAD mice, we performed genotyping using polymerase chain reaction (PCR) for wild-type and mutant embryos. During genotyping procedures, cell viability was maintained by placing embryonic hippocampal tissue in warm Neurobasal medium on a thermoshaker (750 rpm, 37°C).

The 5xFAD mouse line expresses both mutant human APP695, which harbors the Swedish mutation (K670N, M671L), the Florida mutation (I716V), and the London mutation (V717I), and human PSEN1, which harbors two FAD mutations (M146L and L286V). Both transgenes are expressed under the control of the mouse Thy1 promoter to induce overexpression in the brain. Primers for PCR are shown in Table 1. PCR was performed with Taq polymerase in a C-1000 Thermal Cycler (Bio-Rad) with the following steps:

**TABLE 1:** PCR primers for genotyping wild-type and mutant embryos.

Primer type	Name	Sequence
**(A) PSEN1 Primers**		
Transgene	oIMR1644	5′-AAT AGA GAA CGG CAG GAG CA-3′
Transgene	oIMR1645	5′-GCC ATG AGG GCA CTA ATC AT-3′
Internal positive control	oIMR7338	5′-CTA GGC CAC AGA ATT GAA AGA TCT-3′
Internal positive control	oIMR7339	5′-GTA GGT GGA AAT TCT AGC ATC ATC C-3′
**(B) APP Primers**		
Transgene	oIMR3610	5′-AGG ACT GAC CAC TCG ACC AG-3′
Transgene	oIMR3611	5′-CGG GGG TCT AGT TCT GCA T-3′
Internal positive control	oIMR7338	5′-CTA GGC CAC AGA ATT GAA AGA TCT-3′
Internal positive control	oIMR7339	5′-GTA GGT GGA ATT TCT AGC ATC ATC C-3′

Lid: 110° C
Volume: 20 μl
(1). Denaturation: 94°C, 3:00;
(2). Denaturation: 94°C, 0: 30;
(3). Annealing: 64°C, 1:00;
(4). Elongation: 72°C, 1:00;
(5). Beginning at step 2, repeat 35 times;
(6). 72°C, 2:00;
(7). Storage: 4°C, ∞.

PCR was carried out in 0.2-ml disposable tubes with optically transparent caps. The reaction mixture for each gene was prepared in accordance with the following protocol (Table 2):

**TABLE 2:** Reaction/components for 1 sample.

PSEN1	Volume (μl)	APP	Volume (μl)
ddH_2_O	13.2	ddH_2_O	13
5x PCR Buffer	4	5x PCR Buffer	4
dNTP	0.4	dNTP	0.4
oIMR1644	0.3	oIMR3610	0.4
oIMR1645	0.3	oIMR3611	0.4
oIMR7338	0.2	oIMR7338	0.2
oIMR7339	0.2	oIMR7339	0.2
Taq polymerase	0.4	Taq polymerase	0.4

In a test tube, 19.5 μl of the prepared PSEN1 reaction mixture, 0.5 μl of a DNA template sample, 19 μl of the prepared amyloid precursor protein (APP) reaction mixture and 1 μl of a DNA template sample were added.

To discriminate the genotype of individual mice, we used 2% agarose gel electrophoresis.

The expected results for the transgenes were a 610-bp fragment for PSEN1 and 377-bp fragment for APP, with a 325-bp fragment for PSEN1 and 324-bp fragment for APP as the internal positive controls.

### Preparation of Amyloid β Treated With Hexafluoroisopropanol (HFIP)

To prepare 1 mM Aβ, we added HFIP solution (220 μl) directly to the lyophilized β-amyloid peptide powder (Aβ42) (InnovaGen, Sweden) and incubated the mixture at room temperature for 30 min. The obtained solution was transferred to microcentrifuge tubes and left in a hood overnight until a transparent film formed and HFIP evaporated. Next, dimethylsulfoxide (DMSO) was added to the tubes, which were mixed on a vortex for approximately 30 s and then centrifuged at 1000 rpm for 1 min. The Aβ-DMSO solution (5 mM) was placed in a sonicator for 10 min. To prepare a fibrillar β-amyloid peptide, we added 10 mM HCl (98 μL) to the Aβ-DMSO solution, mixed the solution for 15 s and then incubated it at 37°C for 24 h.

### β-Amyloidopathy Model Based on Synthetic Aβ42

We used two protocols for *in vitro* β-amyloidopathy modeling. The first model involved one application of Aβ42 to the culture medium at a final concentration of 3.5 μM on day 10 of primary hippocampal culture development *in vitro* (DIV 10).

**FIGURE 1:**
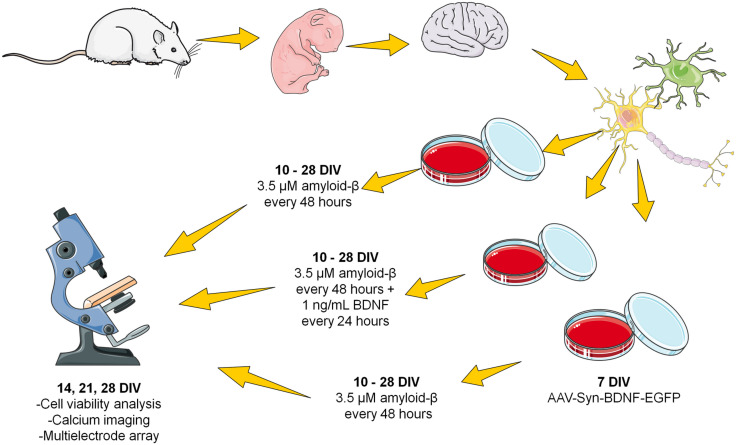
Scheme of the chronic amyloid-β application. The fibrillar amyloid-β was added to the culture medium every 48 h (i.e., after each change of culture medium) at a final concentration of 3.5 μM from DIV 10 to DIV 28.

The second protocol involved chronic application of the same Aβ concentration. The obtained fibrillar amyloid-β was added to the culture medium every 48 h (i.e., after each change of culture medium) at a final concentration of 3.5 μM from DIV 10 to DIV 28 (Figure 1).

### AAV-Syn-BDNF-EGFP Virus Vector

To obtain a viral construct encoding the BDNF gene, we used the following plasmids: AAV-Syn-EGFP and helper plasmids pDP5, DJvector and pHelper. The same plasmids with cDNA EGFP were used to produce a control viral construct - AAV-Syn-EGFP.

The bacterial pUC19 plasmid served as the basis for the AAV-Syn-EGFP plasmid. This plasmid carries the sequences of the human synapsin (hSyn) promoter, woodchuck hepatitis posttranscriptional regulatory element (WPRE) enhancer, and SV40 polyA signal sequence flanked by inverted terminal repeats (ITRs) from adeno-associated serotype 2 virus (AAV2). The developed AAV-Syn-BDNF-EGFP included the following sequences: (1) the hSyn promoter, allowing expression of the gene of interest only in neuronal cells; (2) the regulatory WPRE enhancer, which markedly strengthens hSyn function; (3) a multilinker for open reading frame (ORF) cloning of the embedded gene; (4) the EGFP gene; (5) the SV40 polyA signal sequence flanked by ITRs from AAV2; (6) a gene cassette encoding ampicillin resistance (AmpR promoter and AmpR gene) for positive selection of colonies carrying this plasmid; and (7) a sequence corresponding to the nucleotide sequence encoding the functional BDNF protein.

The designed primers mBDNF-EcoRI-fw (5′-ATTGAATT CATGGGCCACATGCTGTCC-3′) and mBDNF-BamHI-rv (5′-AATGGATCCAATCTTCCCCTTTTAATGGTCAGTG-3′) were used. Detailed procedures for the generation and isolation of the viral construct are described in Mitroshina et al. (2018).

Primary hippocampal cultures were infected with AAV-Syn-BDNF-EGFP or vehicle (AAV-Syn-EGFP) on DIV 7. To infect the cultures, we mixed 3 μL of the viral sample with 50 μL of fresh culture medium. The medium was temporarily removed from the culture dishes, and the working solution of the viral vector was directly added to the cells. The cultures were incubated at 35.5°C with 5% CO_2_ for 20 min; then, the culture medium was returned to the cultures.

### Cell Viability Analysis

To identify dead cell nuclei and the total number of cell nuclei, we stained primary hippocampal cultures with propidium iodide (Sigma, Germany) and bisbenzimide (Thermo Fisher, United States) according to Vedunova et al., 2015. Propidium iodide and bisbenzimide at concentrations of 5 μg/mL and 1 μg/mL, respectively, were added to the culture medium 30 min before viability measurements. Visualization of stained cells was carried out on a Leica DMIL HC inverted fluorescence microscope (Leica, Germany). We estimated the ratio of the number of propidium iodide-positive cells to the number of bisbenzimide-positive cells.

### Immunocytochemical Analysis

The presence of Aβ in dissociated hippocampal cultures was detected by using primary chicken antibodies to Aβ (1:1000, Abcam, ab2539, United Kingdom) and secondary antibodies conjugated to a goat anti-chicken fluorescent marker (1: 100, Alexa Fluor 555, Invitrogen, 1719602, United States). Primary guinea pig antibodies to βIII-tubulin (1:1000, Synaptic systems, 302304, Germany) and secondary antibodies conjugated to a goat anti-guinea pig fluorescent label (1:100, Alexa Fluor 647, Invitrogen, 1711474, United States) were used as neuronal markers. The cultures were fixed in 4% paraformaldehyde in PBS for 20 min at room temperature; 0.2% Triton X-100/PBS was used for cell permeabilization. Immunocytochemically stained cultures were imaged using a Zeiss 510 NLO fluorescent confocal microscope (Carl Zeiss, Germany). The obtained images were analyzed using a custom ImageJ plugin. We conducted a comparative assessment of observation fields with equal densities of cells and imaged by the same laser power and photodetector settings. The average fluorescence intensity in the yellow channel, corresponding to the presence of Aβ42 in the observation field, was estimated.

### Ca^2+^ Imaging

For imaging studies of functional Ca^2+^ activity in primary hippocampal cultures, we used a Zeiss 510 NLO fluorescent confocal microscope (Carl Zeiss, Germany) with a W Plan-Apochromat 20 × /1.0 objective. This method allows visualization of the functional neural network architecture at the cellular level. Oregon Green 488 BAPTA-1 AM (OGB-1) (0.4 μM, Thermo Fisher, United States), which was used as a calcium sensor, was dissolved in DMSO (Sigma, Germany) with 4% Pluronic F-127 (Thermo Fisher, United States) and then added to the culture medium for 40 min at 37°C and 5% CO_2_. OGB-1 was excited at 488 nm and recorded in the range of 500–530 nm. Time series of 512 × 512 pixel images of 420 × 420-μm fields of view were recorded at 2 Hz. A confocal pinhole of 1 airy unit was used to obtain an axial optical slice resolution of 1.6 μm. Detection and further analysis of Ca^2+^ oscillations was performed in the Astroscanner program. A more detailed description of the image analysis is provided in our previous articles (Vedunova et al., 2013; Zakharov et al., 2013). The following parameters of spontaneous Ca^2+^ activity were taken into account: the percentage of functional active cells and the duration (s) and frequency (the amount of Ca^2+^ events/min) of Ca^2+^ oscillations.

### Electrophysiological Methods and Cross-Correlation Analysis

Spontaneous bioelectrical activity of neural networks in primary hippocampal cultures under chronic Aβ application was measured on days 14, 21, and 28 of cultivation. Extracellular action potentials were detected by MEAs (MEA60) and the USB-MEA-120 system (Multichannel system, Germany). The MEAs consisted of 59 planar TIN electrodes 8 × 8 grid) with a diameter of 30 μm and spaced 200 μm apart. Electrophysiological data were recorded simultaneously from 59 channels at a sampling rate of 20 kHz/channel. All signaling and statistical analyses were performed using custom-made software (MATLAB^®^6.0, United States).

Small network bursts were detected by calculating the total spiking rate (TSR), which considered the total number of spikes from all electrodes within 50-ms time bins. The criterion of a small network burst was the rapid appearance of a large number of spikes over four electrodes within a small (50-ms) time bin (Pimashkin et al., 2011; Vedunova et al., 2013). A more detailed description of the method for spikes and small burst detection is provided in our previous article (Mishchenko et al., 2019).

The following parameters of spontaneous bioelectrical activity of neural networks were analyzed: the number of small network bursts and the number of spikes per burst.

For cross-correlation analysis, the dataset obtained from electrophysiological recordings is presented as a raster plot. The network graph method was then used to detect the neuronal groups.

To assess the degree of synchronization between all pairs of cells, considering axonal delays, we calculated the proportion of transmitted spikes. The number of delayed synchronous spikes was normalized by the number of spikes received by the postsynaptic neuron *n*_*j*_. The cross-correlation matrix was calculated using the following formula:

(1) $$C_{i\,j}=\frac{n_{s\,y\,n\,c\,h\,r,i\,j}}{n_{j}}$$

Next, we selected the largest 5% of *C*_*ij*_ coefficients and defined a set of indices, i.e., hubs of cells with a maximum number of functionally active connections. In addition, for each hub “*i*,” we calculated the number of connections to index *i* within the array *C*_*ij*_.

Next, the graph was constructed. The vertex size was proportional to the number of significant connections, and the edge of the graph corresponded to the functional connections of spikes transferred from one neuron to another at individual time points for each pair of axonal delays, i.e., *τ* ± δ/2 (Shishkina et al., 2018).

### Real-Time PCR

Quantitative real-time PCR was used to analyze the levels of TrkB-FL receptor (TrkB gene) expression. Total RNA was isolated from primary hippocampal cell cultures on DIV 21 under chronic Aβ application using an ExtractRNA kit (eUROGEN, Russia). Then, cDNA was synthesized by Moloney murine leukemia virus (MMLV) reverse transcriptase (eUROGEN, Russia) and a random primer.

Quantitative real-time PCR was performed with qPCRmix-HS SYBR (eUROGEN, Russia) and an Applied Biosystems 7500 RT-PCR thermal cycler. The following primers were used:

TrkB-fw1, 5′-TTTCCGCCACCTTGACTTGTCT-3′;
TrkB-rv1, 5′-GTCGGGGCTGGATTTAGTCTCC-3′;
Oaz1_fw, 5′- AAGGACAGTTTTGCAGCTCTCC -3′; and
Oaz1_rv, 5′- TCTGTCCTCACGGTTCTTGGG-3′.

Data processing was carried out using the ΔΔCt method and a reference sample in which the target gene level was taken as a unit. Normalization was performed relative to the reference gene (Oaz1).

### Statistical Analysis

All quantified data are presented as the mean ± standard error of the mean (SEM). Statistical analyses were performed using two-way ANOVA implemented in Sigma Plot 11.0 software (Systat Software, Inc.). The Student–Newman–Keuls (SNK) test was used as a *post hoc* test following ANOVA. Differences between groups were considered significant if the corresponding *p*-value was less than 0.05.

## Results

### Features of the Morphology and Spontaneous Calcium Activity of Neuron-Glial Networks in Primary Hippocampal Cultures Obtained From 5xFAD Murine Embryos

First, we adapted an *in vitro* amyloidopathy model, allowing studies of changes in neural network activity. To obtain a valid model, we conducted a single and chronic application of Aβ1-42 to primary hippocampal cultures obtained from C57BL/6 murine embryos and investigated the features of long-term cultivation of primary hippocampal cultures obtained from 5xFAD murine embryos.

**FIGURE 2:**
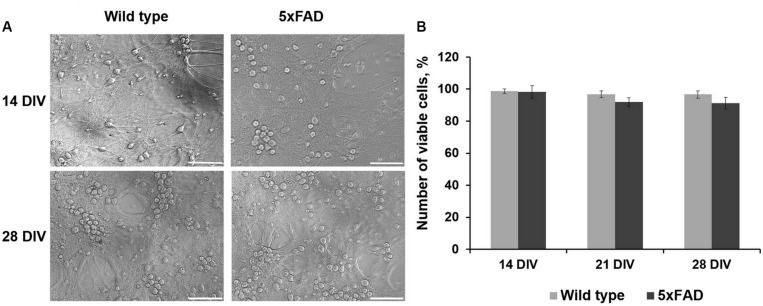
**(A)** Representative light field images of primary hippocampal cultures obtained from wild-type and 5xFAD mice. Scale bar – 100 μm. Comparative morphological assessment did not reveal significant changes between primary hippocampal cultures obtained from wild-type and 5xFAD murine embryos over 28 DIV. **(B)** Analysis of cell viability in primary hippocampal cultures obtained from wild-type and 5xFAD murine embryos. There was no decrease in cell viability in either experimental group.

Comparative morphological assessment did not reveal significant changes between primary hippocampal cultures obtained from wild-type and 5xFAD murine embryos over 28 DIV (Figure 2, Supplementary Figure S1). There was also no decrease in cell viability in either experimental group (Figure 2).

**FIGURE 3:**
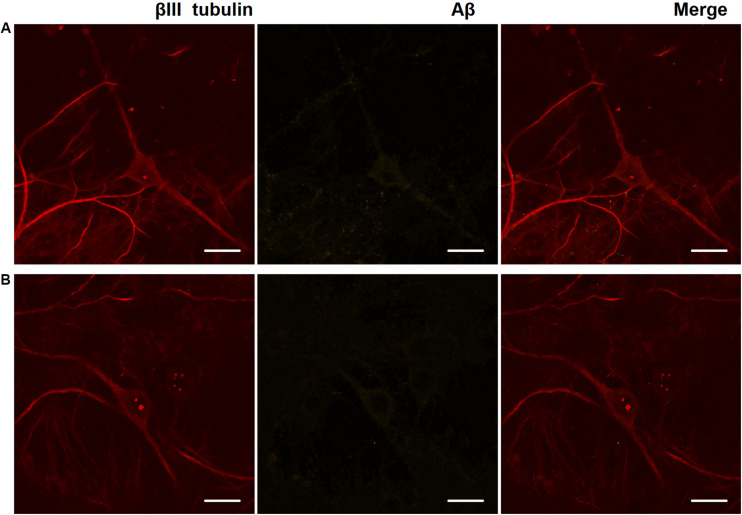
Representative confocal images of immunocytochemical staining of primary hippocampal cultures on DIV 28. **(A)** Primary hippocampal cultures obtained from C57BL/6 murine embryos. **(B)** Primary hippocampal cultures obtained from 5xFAD murine embryos. Red: fluorescence channel of βIII tubulin (neuronal microtubule marker); yellow: fluorescence channel of Aβ. Scale bar – 20 μm. The level of Aβ expression did not change significantly.

Immunocytochemical analysis of Aβ accumulation in primary hippocampal cells obtained from C57Bl/6 and 5xFAD mice revealed that both types of cultures underwent endogenous Aβ synthesis, which was detected intraneuronally. The level of Aβ expression did not change significantly throughout the entire observation period (DIV 14, 17, 21, and 28) (Figure 3). Thus, an increase in the production of endogenous amyloid in the 5xFAD murine brain may occur in later stages. Numerous studies have shown that amyloid plaques are formed in the 5xFAD murine brain after eight months of age, although the first cognitive impairments are observed beginning at four months of age (Radde et al., 2006; Bilkei-Gorzo, 2014; Liu et al., 2017).

**FIGURE 4:**
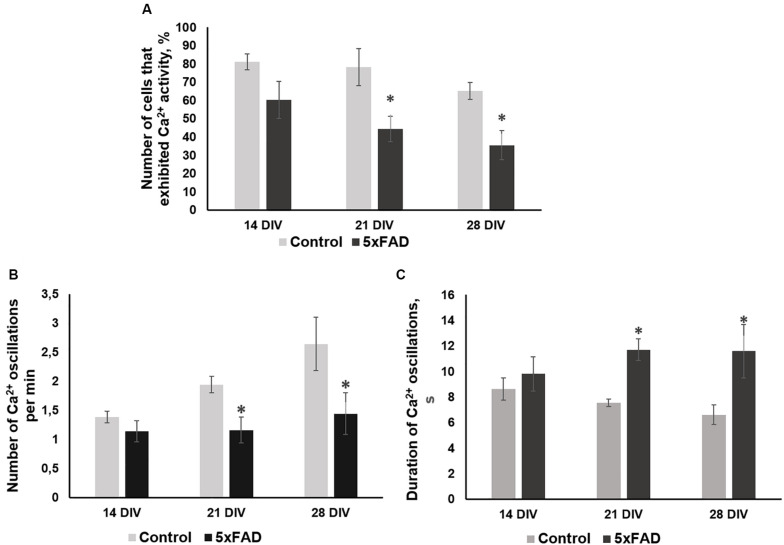
Main parameters of spontaneous calcium activity in primary hippocampal cultures obtained from wild-type and 5xFAD murine embryos (E18) during development *in vitro*. **(A)** Proportion of cells exhibiting Ca^2+^ activity; **(B)** number of Ca^2+^ oscillations per min; **(C)** duration of Ca^2+^ oscillations. ^∗^vs. “Control,” *p* < 0.05, ANOVA. The functional Ca^2+^ activity of 5xFAD primary neuronal cultures was significantly altered in comparison with that of wild-type murine primary cultures. The percentage of cells that exhibited Ca^2+^ activity and the frequency of Ca^2+^ oscillations in the cultures obtained from 5xFAD murine embryos were significantly lower than that in control cultures. Additionally, the duration of Ca^2+^ oscillations in 5xFAD primary cultures was 1.55 (DIV 21) and 1.75 (DIV 28) times higher than those in control cultures.

No morphological changes in primary 5xFAD murine hippocampal cultures were shown, and Aβ accumulations were not observed. However, the functional Ca^2+^ activity of primary 5xFAD murine hippocampal cultures was significantly altered in comparison with that of neuronal cultures obtained from wild-type murine embryos (Figure 4). Spontaneous network Ca^2+^ activity was detected beginning at DIV 10, consistent with previous data on primary hippocampal culture development *in vitro* (Shirokova et al., 2013). However, the percentage of cells that exhibited Ca^2+^ activity in the cultures obtained from 5xFAD murine embryos was significantly lower than that in the control cultures (DIV 21: control, 78.2 ± 10.02%; 5xFAD, 44.3 ± 7.02%; DIV 28: control, 65 ± 4.6%; 5xFAD, 35.5 ± 7.91%). The frequency of Ca^2+^ oscillations in the 5xFAD group of cultures was also significantly lower than the control values (DIV 21: control, 1.94 ± 0.14 oscillations/min (osc/min); 5xFAD, 1.16 ± 0.22 osc/min; DIV 28: control, 2.64 ± 0.46 osc/min; 5xFAD, 1.44 ± 0.36 osc/min). Additionally, the duration of Ca^2+^ oscillations in 5xFAD primary cultures was 1.55 (DIV 21) and 1.75 (DIV 28) times higher than those in the control cultures. The identified alterations in spontaneous calcium activity of 5xFAD primary cultures in the absence of pronounced morphological changes suggest that functional changes in nervous cells occur much earlier than visible neurodegenerative changes.

### Features of the Morphology and Spontaneous Calcium Activity of Neuron-Glial Networks in Primary Hippocampal Cultures in the β-Amyloidopathy Model *in vitro*

Next, we used an *in vitro* β-amyloidopathy model established by adding synthetic Aβ1-42 to the culture medium. Two amyloidopathy modeling protocols were adapted, with the first involving one application of Aβ at a final concentration of 3.5 μM on DIV 10, and the second (chronic application) involving the introduction of Aβ to the culture medium at a final concentration of 3.5 μM every 48 h (i.e., after each change of culture medium) from DIV 10 to DIV 28. Notably, DIV 10 is a time of culture development characterized by a large number of chemical synapses and the formation of spontaneous bioelectrical and calcium network activity (Shirokova et al., 2013).

**FIGURE 5:**
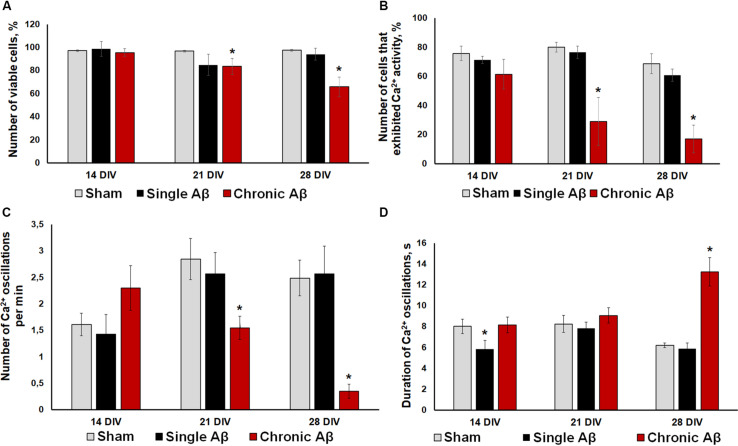
**(A)** Analysis of cell viability in primary hippocampal cultures under single and chronic exogenous application of Aβ. * vs. “Sham,” *p* < 0.05, ANOVA. One application of Aβ did not affect primary culture viability, whereas its chronic application significant decreased the number of viable cells. **(B–D)** Main parameters of spontaneous calcium activity in primary hippocampal cultures under single and chronic Aβ application **(B)** Proportion of cells exhibiting Ca^2+^ activity; **(C)** number of Ca^2+^ oscillations per min; **(D)** duration of Ca^2+^ oscillations. * vs. “Sham,” *p* < 0.05, ANOVA. One Aβ application did not cause a pronounced effect on Ca^2+^ activity in neuron-glial networks. Severe Ca^2+^ activity suppression under chronic Aβ application was observed from DIV 21.

**FIGURE 6:**
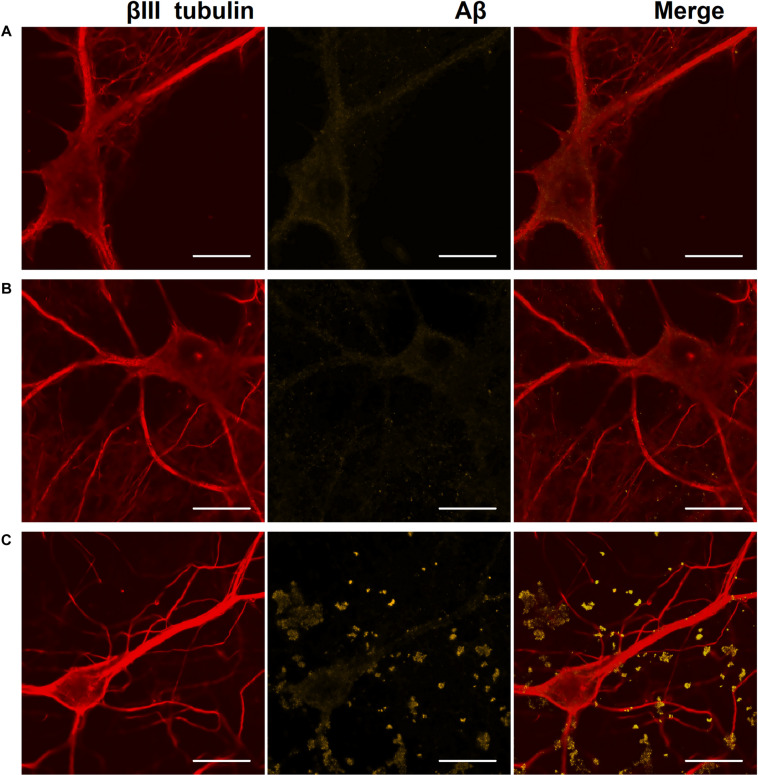
Representative confocal images of immunocytochemical staining of primary hippocampal cultures on DIV 28. **(A)** Sham culture, **(B)** primary culture with single Aβ application, **(C)** primary culture with chronic Aβ application. Red: fluorescence channel of βIII tubulin (neuronal microtubule marker); yellow: fluorescence channel of Aβ. Scale bar – 20 μm. A significant amount of Aβ associated with cells as protein globules was detected only in cultures with chronic Aβ application.

To verify the effectiveness of the experimental protocols used, we performed a cell viability assessment (Figure 5A) and immunohistochemical analysis of Aβ aggregate formation in primary hippocampal cultures (Figure 6). One application of Aβ did not affect primary culture viability. By contrast, chronic application of Aβ led to a significant decrease in the number of viable cells. On DIV 21, the percentage of living cells in the chronic Aβ group was 83.57 ± 6.95% (sham: 96.74 ± 0.74%) and continued to decrease at DIV 28 (sham: 97.47 ± 0.62%, chronic Aβ: 65.87 ± 8.39%).

On DIV 28, immunocytochemical analysis revealed a significant amount of Aβ associated with cells as protein globules only in cultures with chronic Aβ application (Figure 6).

Moreover, the effect of Aβ application using the two protocols on functional calcium activity of neuron-glial networks in primary hippocampal cultures was studied. In addition to the lack of influence on cell viability, one Aβ application did not cause a pronounced effect on Ca^2+^ activity in neuron-glial networks. No significant changes in the number of cells exhibiting Ca^2+^ activity or the frequency of Ca^2+^ oscillations throughout the observation period were revealed (Figures 5B–D). The detected decrease in the duration of Ca^2+^ oscillations on DIV 14 (sham: 8.03 ± 0.73 s, single Aβ: 5.84 ± 0.85) was normalized to sham values by DIV 21 (sham: 8.26 ± 0.81 s, single Aβ: 7.82 ± 0.61).

Severe Ca^2+^ activity suppression under chronic Aβ application was observed from DIV 21, consistent with the cell viability analysis. Compared to the sham group, the chronic Aβ group had a significantly lower number of cells that exhibited Ca^2+^ activity on DIV 21 and DIV 28 (DIV 21: sham, 80.06 ± 3.40%; chronic Aβ, 29.01 ± 16.38%; DIV 28: sham, 68.70 ± 9.53%; chronic Aβ, 17.18 ± 9.48%). On DIV 28, the chronic Aβ group exhibited a significantly lower Ca^2+^ oscillation frequency (sham: 2.49 ± 0.34 osc/min, chronic Aβ: 0.35 ± 0.13 osc/min) and a significantly higher Ca^2+^ oscillation duration (sham: 6.2 ± 0.23 s, chronic Aβ: 13.24 ± 2.37 s) than the sham group.

In further studies, we used the AD model based on chronic Aβ application because it caused the most pronounced neurodegenerative changes.

### Effects of Chronic BDNF Application on Cell Viability, Bioelectrical and Calcium Activity of Primary Hippocampal Cultures in the Chronic β-Amyloidopathy Model

**FIGURE 7:**
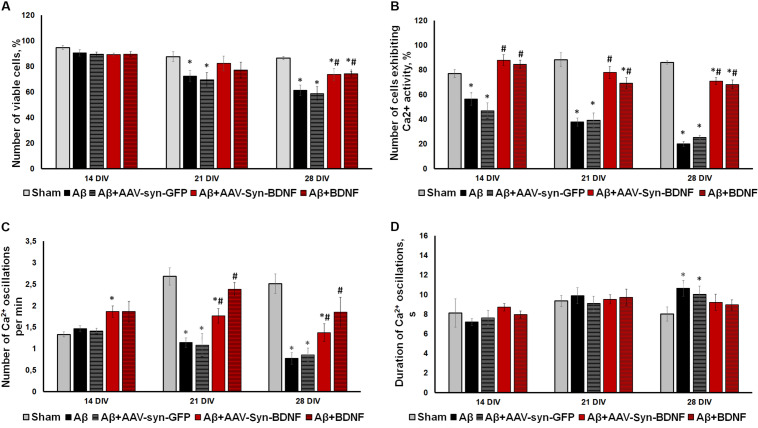
**(A)** Analysis of cell viability in primary hippocampal cell cultures under exogenous administration of Aβ and BDNF. * vs. “Sham,” ^#^ vs. “Aβ,” *p* < 0.05, ANOVA. On DIV 21 and 28 when Aβ and AAV-Syn-EGFP application caused a significant decrease in culture viability, both recombinant BDNF and AAV-Syn-BDNF preserved the number of viable cells. **(B–D)** Main parameters of spontaneous calcium activity in primary hippocampal cell cultures under chronic exogenous application of Aβ and BDNF during development *in vitro*. **(B)** Proportion of cells exhibiting Ca^2+^ activity; **(C)** number of Ca^2+^ oscillations per min; **(D)** duration of Ca^2+^ oscillations. ^∗^vs. “Sham,” ^#^ vs. “Aβ,” *p* < 0.05, ANOVA. On DIV 28, the activity of cells in BDNF-treated cultures was significantly higher compared to those in cultures exposed to chronic Aβ. The frequency of Ca^2+^ oscillations in the Aβ+BDNF and Aβ+AAV-Syn-BDNF groups was significantly higher than that in the Aβ group. Moreover, the duration of Ca^2+^ oscillations in the BDNF-treated group did not differ from that in the sham group on DIV 28. The parameters of spontaneous calcium activity in the “Aβ+AAV-Syn-EGFP” group did not differ from the values in the “Aβ” group.

To investigate the possible neuroprotective effect of chronic BDNF application in our amyloidopathy model, we analyzed primary hippocampal cell viability. On DIV 21, when Aβ application caused a significant decrease in culture viability, both recombinant BDNF and AAV-Syn-BDNF preserved the number of viable cells (sham: 87.63 ± 3.72%, Aβ: 72.52 ± 4.18%, Aβ+BDNF: 77.11 ± 6.22%, Aβ+AAV-Syn-BDNF: 82.34 ± 5.60%). The use of control virus vector AAV-Syn-EGFP did not preserve the viability of neuronal cultures. The viability parameter did not differ from that in the Aβ group and was significantly lower than the values in the sham group (Aβ+AAV-Syn-EGFP: 69.41 ± 5.87%). On DIV 28, the percentage of viable cells in the Aβ and Aβ+AAV-Syn-EGFP groups was decreased to 61.34 ± 3.38% and 58.72 ± 5.31% respectively, whereas in the Aβ+BDNF and Aβ+AAV-Syn-BDNF groups, this parameter was significantly higher at 76.96 ± 2.20% and 72.01 ± 2.95%, respectively (Figure 7A).

Analysis of the main parameters of spontaneous Ca^2+^ activity identified significant changes in the functional state of neuronal cells beginning at DIV 21. The number of cells that exhibited Ca^2+^ activity in the Aβ+BDNF and Aβ+AAV-Syn-BDNF groups was significantly higher than that in the Aβ and Aβ+AAV-Syn-EGFP groups (DIV 21: sham: 88.27 ± 5.56%, Aβ: 37.74 ± 3.27%, Aβ+AAV-Syn-EGFP: 39.21 ± 5.97%; Aβ+AAV-Syn-BDNF: 77.83 ± 4.89%, Aβ+BDNF: 69.31 ± 4.35%). On DIV 28, the neuroprotective effect remained; the activity of cells in primary cultures treated with BDNF was significantly higher than that of those in AAV-Syn-EGFP-treated cultures and in cultures exposed to chronic Aβ (Figures 7B–D).

A decreased frequency of Ca^2+^ oscillations and an increased duration of Ca^2+^ events caused by chronic Aβ application were observed on DIV 21. Additionally, the frequency of Ca^2+^ oscillations in the Aβ+BDNF and Aβ+AAV-Syn-BDNF groups was significantly higher than that in the Aβ group group (DIV 21: Aβ, 1.14 ± 0.11 osc/min; Aβ+AAV-Syn-BDNF, 1.76 ± 0.17 osc/min; Aβ+BDNF, 2.38 ± 0.16 osc/min). The frequency of Ca2+ oscillations in the Aβ+AAV-Syn-EGFP group did not differ from that in the Aβ group (1.08 ± 0.27 osc/min). Moreover, the duration of Ca^2+^ oscillations in the BDNF-treated group did not differ from that in the sham group on DIV 28 (Figures 7C,D).

Thus, an AAV construct containing the BDNF gene, but not an AAV-Syn-EGFP virus vector, prevented impairments in functional neural network Ca^2+^ activity in our amyloidopathy model *in vitro*. The effect of BDNF hyperexpression was comparable to the effects of chronic recombinant protein application.

Electrophysiological data analysis revealed that Aβ disrupted neural network formation on DIV 14, and this effect was characterized by a significant decrease in the number of spikes in a small network burst (Table 3).

**TABLE 3:** Main parameters of spontaneous bioelectrical activity in primary hippocampal cell cultures under chronic Aβ application.

Day of cultivation	Sham	Aβ	Aβ+BDNF	Aβ+AAV-syn-BDNF
**(A) Number of small network bursts/5 min**				
DIV14	163.228.5	112.831.92	142.132.21	97.635.43
DIV 21	217.535.8	74.320.8*	131.823.54*^#^	117.924.29*
DIV 28	253.741.3	50.114.71*	175.535.2^#^	147.342.3*^#^
**(B) Number of spikes per burst**				
DIV 14	382.756.9	149.142.2*	16331.48*	17251.2*
DIV 21	189.534.7	68.113.2*	15740.2^#^	16438.4^#^
DIV 28	238.524.6	136.131.9*	187.239.6^#^	200.447.2^#^
**(C) Number of large network bursts/10 min in primary hippocampal cell cultures under chronic Aβ application**				
DIV 14	12.58.3	0*	0*	0*
DIV 21	17.95.8	0*	10.25.3	15.14.9
DIV 28	15.44.3	224.7	13.14.8	16.54.2

*vs. sham, *p* < 0.05; ^#^vs. Aβ, *p* < 0.05; ANOVA, *N* = 3.

Further suppression of spontaneous bioelectrical activity in the cultures with chronic Aβ application was observed at a later stage. Compared to the sham group, the Aβ group exhibited fewer small network bursts and spikes in a burst on DIV 21 and DIV 28 (Table 3). BDNF partially negated the decreased spontaneous bioelectrical activity of primary hippocampal cultures. On DIV 21, the number of small network bursts in the Aβ+BDNF group was significantly higher than that in the Aβ group. Moreover, the number of spikes in a network burst in the Aβ+BDNF and Aβ+AAV-Syn-BDNF groups did not differ from that in the sham group (Table 3).

According to the classical concept, a network burst is an event comprising no fewer than four spikes simultaneously recorded from different electrodes in a 50-ms interval (Wagenaar et al., 2006; Pimashkin et al., 2011; Vedunova et al., 2013). However, for a more detailed analysis of the complex structure of the neural network, we selected events that simultaneously captured the prevailing part of functionally active cells. In this regard, all neural network bursts were conditionally divided into small (from 4 to 100 spikes in 50 ms) and large (101 or more spikes in 50 ms) groups (Mishchenko et al., 2019).

**FIGURE 8:**
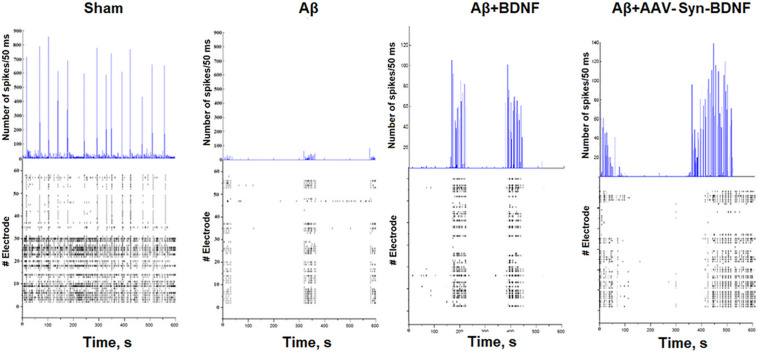
Number of spikes/50 ms and representative raster diagrams of spontaneous bioelectrical activity in primary hippocampal cultures under chronic Aβ application (DIV 21). The use of Aβ completely inhibited large network burst formation in cultures until DIV 28. In contrast, in cultures treated with recombinant BDNF and AAV-syn-BDNF the formation of complex network bursts were observed on DIV 21.

Our studies revealed that Aβ almost completely inhibited large burst formation in primary hippocampal cultures at early stages (Table 3C, Figure 8). Large network bursts were first measured in the sham group at DIV 14, whereas large network events in the Aβ group were completely absent on both DIV 14 and DIV 21 and did not begin to form until DIV 28. However, the formation of large network bursts was in cultures treated with recombinant BDNF and AAV-Syn-BDNF on DIV 21.

**FIGURE 9:**
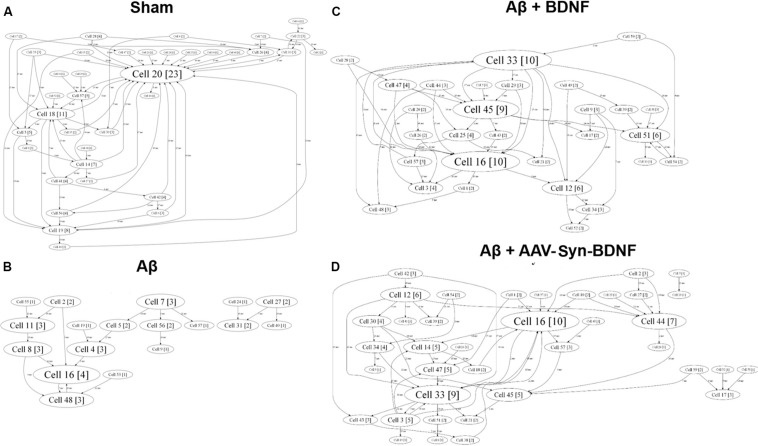
Internal functional structure of neural networks in the primary hippocampal cultures on DIV 21. Graphical representation of the correlated connections among neurons in the network. The electrode number is presented as “Cell X.” The number of connections on the electrode is indicated in square brackets. The vertex size is proportional to the number of significant connections. **(A)** Sham; **(B)** Aβ; **(C)** BNDF; **(D)** Aβ+AAV-syn-BDNF. A neuronal network in an intact primary hippocampal culture has a complex architecture, which includes several active hubs. Chronic application of synthetic Aβ simplified the internal neural network structure. BDNF preserved the complexity of neural network architecture and maintained the active centers in the network throughout the entire observation period.

For a better understanding of neural network structure, we performed a cross-correlation analysis and correlation graph reconstruction to study the structure of functional interconnections in a network with a definition of its key activity elements–hubs (Shishkina et al., 2018). On DIV 14, a neuronal network in an intact primary hippocampal culture has a complex architecture, which includes several active hubs that form at least 10 connections between nearby electrodes (Figure 9). AD modeling by chronic application of synthetic Aβ led to significant simplification of the internal neural network structure on DIV 14. At this time, the number of connections between the network elements was 5 times lower than that in the sham group (Table 4). Simplification of the internal functional structure of neural networks in the Aβ group continued during culture development (Figure 9). Active centers and elements forming at least 10 connections were nearly absent on DIV 21. By DIV 28, neural network activity was almost completely abolished.

**TABLE 4:** Average number of connections in a hub in primary hippocampal neural networks on different days of development *in vitro.*

Day of cultivation	Sham	Aβ	Aβ+BDNF	Aβ+AAV-syn-BDNF
DIV 14	11.2 ± 2.8	2.2 ± 1.6*	12.4 ± 3.2^#^	8.9 ± 2.8^#^
DIV 21	16.5 ± 4.3	1.1 ± 1.3*	12.25 ± 4.1^#^	11.4 ± 3.2^#^
DIV 28	12.6 ± 3.9	0*	12.31 ± 3.7^#^	13.5 ± 4.6^#^

*vs. sham, *p* < 0.05; ^#^vs. Aβ, *p* < 0.05; ANOVA, *N* = 3.

Brain-derived neurotrophic factor preserved the complexity of neural network architecture. The active centers in the network were maintained throughout the entire observation period. The number of connections in the hubs in the BDNF-treated groups did not differ from the values in the sham group (DIV 21: sham, 16.5 ± 4.3; Aβ, 1.1 ± 1.3; Aβ+BDNF, 2.25 ± 4.1; Aβ+AAV-Syn-BDNF, 11.4 ± 3.2).

Next, we assumed that a molecular mechanism of BDNF neuroprotective action could be associated with activation of TrkB receptors, having a high affinity to BDNF. For this purpose, using RT-PCR, we performed quantitative evaluation of TrkB-FL receptors. We showed no significant alterations in TrkB-FL mRNA levels in primary hippocampal cultures on DIV21 under chronic Aβ1-42 application. The obtained data are presented in Supplementary Figure S2.

## Discussion

Currently, animal models of AD are commonly used to study various changes associated with neurodegenerative processes. However, the ongoing *in vivo* studies do not allow for in-depth analysis of functional neural network activity. There have been a few experimental studies on neuronal cultures obtained from 5xFAD transgenic mice (ex. Park et al., 2016), but they did not investigate the features of functional neuron-glial network activity during AD development. Our data on functional calcium activity inhibition during the cultivation of primary hippocampal cells obtained from 5xFAD murine embryos are of interest since the proposed model may serve as a way to assess functional changes and identify mechanisms of neural network reorganization at the earliest stages of a familial form of AD. However, our model is not suitable for studying the effect of amyloidopathy.

In this study, we used experimental model of amyloidopathy to investigate the functional activity of neural networks *in vitro*. Amyloid protein oligomers are currently considered the most neurotoxic in AD; their accumulation is much better correlated with the severity of cognitive symptoms than the presence of plaques or neurofibrillary tangles (DiChiara et al., 2017; Bourdenx et al., 2017; Cline et al., 2018). Amyloid plaques mainly consist of aggregated Aβ, the most common forms of which are Aβ1-40 and Aβ1-42 (Scarano et al., 2016; Cline et al., 2018). Aβ accumulation initially occurs intraneuronally, mainly in synapses (Pignataro and Middei, 2017; Forner et al., 2017); plaques and tangles form in the brain parenchyma at later stages. Therefore, in our amyloidopathy model, we used Aβ1-42. We demonstrated neurodegenerative effects in this model, characterized by an increase in cell death, and immunocytochemically confirmed the formation of extracellular amyloid aggregates.

A pronounced suppressive action of chronic amyloidopathy on spontaneous functional Ca^2+^ activity in primary hippocampal cultures was also shown. The negative effect of Aβ was demonstrated by decreases in the number of cells exhibiting Ca^2+^ activity and frequency of Ca^2+^ oscillations. Studies on the features of Ca^2+^ network activity in AD are currently limited. Previous *in vitro* experiments have shown that Aβ oligomers can increase cytosolic Ca^2+^ levels, eventually leading to mitochondrial Ca^2+^ overload and partial neuronal death (Kuchibhotla et al., 2008; Villalobos et al., 2012; Tu et al., 2014). Interestingly, this effect of Aβ42 oligomer application was not revealed at the early stages of primary neuronal culture development (2-10 DIV), but Ca^2+^ activity was sharply increased beginning at DIV 14, at which point different age-related signs were observed in neurons. In this regard, the cytosolic and mitochondrial Ca^2+^ responses induced by single Aβ42 oligomer administration increased dramatically over time (adult cultures DIV 21-28) (Calvo-Rodríguez et al., 2016; Núñez et al., 2018). Our data indicated that chronic Aβ42 application increased the suppression of functional Ca^2+^ activity in primary hippocampal cultures and may correlate with *in vivo* studies of Ca^2+^ activity, indicating neural dysfunction in the brains of aging mice with modeled AD. These pathological changes result in the silencing of some neurons, whereas other neurons in areas enriched with Aβ plaques are hyperactive (Busche et al., 2012; Busche, 2018).

Currently, many research groups are interested in calcium activity changes during AD. However, there is still very limited data on the molecular mechanisms underlying these changes. One possible neurodegenerative mechanism in AD is through the hyperstimulation of glutamate receptors, mainly NMDA, resulting in an excessive increase in Ca2+ levels, causing excitotoxicity and further neuronal death (Kabir et al., 2019). Moreover, because numerous studies have demonstrated that Aβ protein oligomer stimulates calcium influx via the NMDA receptor in the pathogenesis of AD, NMDA receptor antagonists are considered potential therapeutic substances for presymptomatic AD (Kodis et al., 2018; Müller et al., 2018). Increased IP3R function is another expected cause of changes in intracellular transmission of Ca^2+^ signals (Mak et al., 2015). Overall, the aspect of calcium activity changes are extremely interesting and merit further detailed investigation.

We also studied the neuroprotective effects of the neurotrophic factor BDNF. We were especially interested in examining these effects in a model that permitted the evaluation of rapid neurodegeneration. Therefore, we used the model based on chronic Aβ application.

Cognitive dysfunction in AD is associated with impairments in neurotrophic factors [such as BDNF, glial cell line-derived neurotrophic factor (GDNF), and nerve growth factor (NGF)] levels in the blood (Budni et al., 2015). Yasutake et al., 2006, showed a decrease in BDNF levels in blood in the late stages of AD. Additionally, Aβ has been shown to directly inhibit BDNF proteolysis from pro-BDNF (Zheng et al., 2010) and reduce the retrograde axonal transport of the BDNF/TrkB system through a mechanism including ubiquitin carboxy-terminal hydrolase L1 (Poon et al., 2013). Thus, a decrease in BDNF levels is likely one of the important molecular mechanisms of AD pathogenesis. In this regard, we considered BDNF a potential neuroprotectant in amyloidosis-induced neurodegenerative processes.

As neurodegeneration has a chronic irreversible pattern in AD, we studied the effects of constant BDNF hyperexpression induced by an AVV vector carrying the BDNF gene. Chronic application of recombinant BDNF protein (1 ng/ml) served as a positive control. Similar viral constructs carrying neurotrophic factor genes are considered promising agents that are being actively studied as gene therapies for neurodegenerative diseases, such as Parkinson’s disease (Cheng et al., 2018; Tereshchenko et al., 2014). However, there is a lack of data on the effectiveness of these approaches in AD. For instance, using a transgenic model of amyloidosis *in vivo*, the team led by Prof. M.H. Tuszynski has shown that viral delivery of BDNF gene after the beginning of pathological processes development restrains the loss of synapses, partially normalizes the aberrant expression of the Aβ gene, improves synaptic transmission and restores learning and memory (Nagahara et al., 2009). Jiao SS. et al. demonstrated the influence of AAV-BDNF construct application *in vivo* (Jiao et al., 2016). The recovery of the BDNF level attenuated behavioral impairments and prevented neuronal loss but did not affect the level of tau hyperphosphorylation in P301L mouse brains. These data indicate that delivery of the BDNF gene is a promising method for neurodegeneration therapy. However, there have been no studies of neural network activity following treatment with genetically engineered constructs in AD.

Our *in vitro* studies using a β-amyloidopathy model showed a pronounced neuroprotective effect of BDNF. Notably, we reported cell viability preservation as well as maintenance of the functional integrity of neural networks, characterized by the normalization of spontaneous Ca^2+^ activity.

Electrophysiological data analysis revealed significant impairments in primary hippocampal neural network activity under chronic Aβ application. Our previous studies have shown several stages of functional activity formation in neuronal cultures. The first single small network bursts are detected on DIV 7; their numbers and the number of spikes in a burst gradually increase by DIV 14. Stable spontaneous bioelectrical activity in primary hippocampal cultures is measured beginning at DIV 14 (Shirokova et al., 2013; Mishchenko et al., 2019).

Our present study revealed that Aβ decreased the number of small network bursts and spikes in a burst on DIV 14 and resulted in a lack of large network events up to DIV 28. A significant simplification in the functional neural network architecture was also observed. These changes are potentially associated with neurodegenerative processes that occur in primary neuronal cultures, with a reduction in cellular outgrowth and synaptic loss, as well as the death of some functionally significant network elements. Changes in spontaneous bioelectrical neural network activity in AD have been very poorly investigated. The available data were mainly obtained by the patch-clamp method and describe the features of synaptic transmission in individual neurons and synaptic endings (Lasala et al., 2019; Mondragón-Rodríguez et al., 2018), and studies using multichannel electroencephalography only indirectly describe neural network activity in AD models (Ahnaou et al., 2017).

Therefore, the electrophysiological data presented in the manuscript characterizing the network activity and functional architecture of neural networks in the AD model are of great interest and may be considered a fundamental basis for the further study of neural network activity, including the application of different stimulation protocols.

A recently published study based on MEA recordings showed that one application of Aβ oligomers initially led to activation followed by further (after 12-24 h) suppression and desynchronization in neural network activity, ultimately resulting in network destruction (Gao et al., 2019). In our work, we studied more prolonged effects caused by amyloid on neural network activity and identified a significant decrease. Our results are consistent with previous data from *in vivo* experiments and studies performed on brain slices obtained from different AD animal models, indicating that the early preclinical stages of AD are characterized by hyperactivation of neuronal activity, whereas the later stages are characterized by its suppression (de Haan et al., 2017; Heggland et al., 2019).

In could be assumed that activation of TrkB receptors is a backbone pathway for BDNF neuroprotective action, and alterations in receptors functions are one of the pathogenetic aspects of AD. A number of studies have shown a decrease in TkrB-FL expression in both *in vitro* and *in vivo* models of AD (Kemppainen et al., 2012; Lei et al., 2018). For instance, rat intracerebroventricular injection of Aβ25-35 solution (5 μL) was shown to result in a significant decrease in TrkB levels (Wang K. et al., 2018). Immunohistochemical studies of postmortem tissues showed a significantly lower staining density of TrkB receptor in the hippocampus of patients with AD than in that of healthy controls. In addition, TrkB staining was inversely correlated with both Amylo-Glo and pTau staining in the same region. These observations strongly confirm that changes in the BDNF-TrkB system are involved in the pathology of AD (Bharani et al., 2019). However, we showed no significant alterations in TrkB-FL mRNA levels in primary hippocampal cultures 2 weeks after the beginning of Aβ application.

In summary, in this work, we examined two experimental models of amyloidopathy using primary hippocampal cultures, one employing chronic application of Aβ1-42 and the other using the embryonic brains of 5xFAD mice. Chronic application of Aβ1-42 resulted in the rapid establishment of significant neurodegenerative changes in primary hippocampal cultures, leading to marked impairments in neural network calcium activity and increased cell death. We studied the influence of amyloidopathy on spontaneous bioelectrical neural network activity in primary hippocampal cultures. Chronic Aβ application led to a decrease in the number of network bursts and spikes in a burst and disturbed the spatial structure of neural networks, reducing the number of key network elements (hubs) and the number of connections between network elements. The model based on primary hippocampal cells obtained from 5xFAD mice demonstrated changes in spontaneous network calcium activity characterized by a decrease in the number of cells exhibiting Ca^2+^ activity, a decrease in the frequency of Ca^2+^ oscillations and an increase in the duration of Ca^2+^ events beginning at DIV 21.

Moreover, application of BDNF recombinant protein and BDNF hyperexpression by an AAV vector partially prevented these amyloidopathy-induced neurodegenerative phenomena. BDNF maintained cell viability and spontaneous bioelectrical and calcium network activity in primary hippocampal cultures. The internal functional structure of neural networks, including the number of hubs and connections between active elements in the network, was also partially preserved.

## Data Availability Statement

The datasets generated for this study are available on request to the corresponding author.

## Ethics Statement

The animal study was reviewed and approved by the Bioethics Committee of Lobachevsky University.

## Author Contributions

EM, TM, and RY carried out the experiments on primary cultures with support from VK. EM and MV wrote the manuscript with input from all other authors. AB and MG designed and developed the virus. EM, TM, and MV ensured the financing of the project. MV supervised the project, conceptualized the original idea, and was in charge of the overall direction of the study. EE performed Real-Time PCR analysis. All authors read and approved the final manuscript.

## Conflict of Interest

The authors declare that the research was conducted in the absence of any commercial or financial relationships that could be construed as a potential conflict of interest.

## Supplementary Material

FIGURE S1 Representative light field images of primary hippocampal cultures obtained from wild-type and 5xFAD murine embryos (DIV 21), Scale bar – 20 μm. Comparative morphological assessment did not reveal significant changes between cultures obtained from wild-type and 5xFAD murine embryos over 28 DIV.

FIGURE S2 Features of TrkB-FL gene expression on DIV 21 under chronic exogenous administration of Aβ and BDNF. Data are normalized to the reference gene (Oaz1). The data represent the mean values ± SEMs from three independent experiments. We showed no significant alterations in TrkB-FL mRNA levels in primary hippocampal cultures on DIV21 under chronic Aβ application.
