# Supplementary figures and images for: Brain-Derived Neurotrophic Factor (BDNF) Preserves the Functional Integrity of Neural Networks in the β-Amyloidopathy Model in vitro
Source: Front Cell Dev Biol. 2020 Jul 8;8:582. doi: 10.3389/fcell.2020.00582 (PMC7360686; doi:10.3389/fcell.2020.00582)

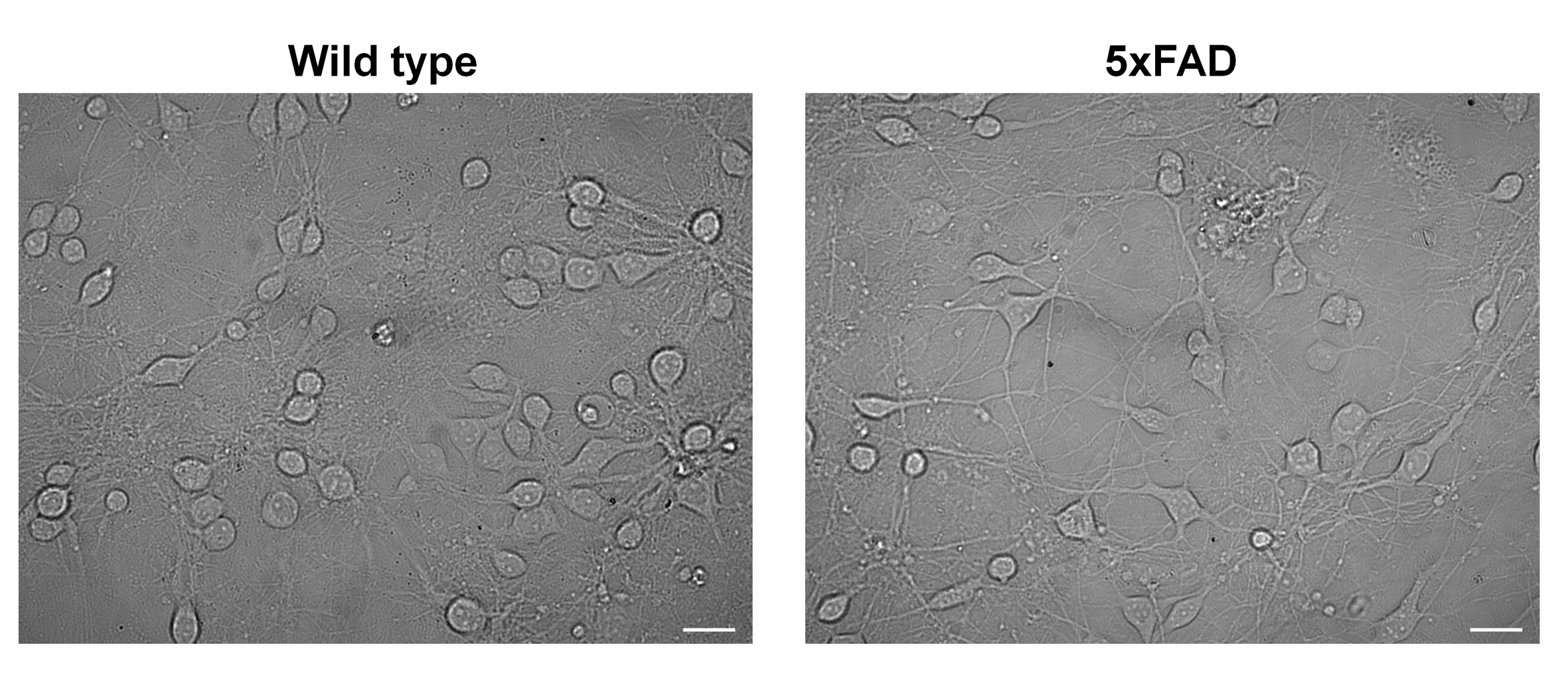

Supplement: FIGURE S1 — Representative light field images of primary hippocampal cultures obtained from wild-type and 5xFAD murine embryos (DIV 21), Scale bar – 20 μm. Comparative morphological assessment did not reveal significant changes between cultures obtained from wild-type and 5xFAD murine embryos over 28 DIV. [file Image_1.TIF]

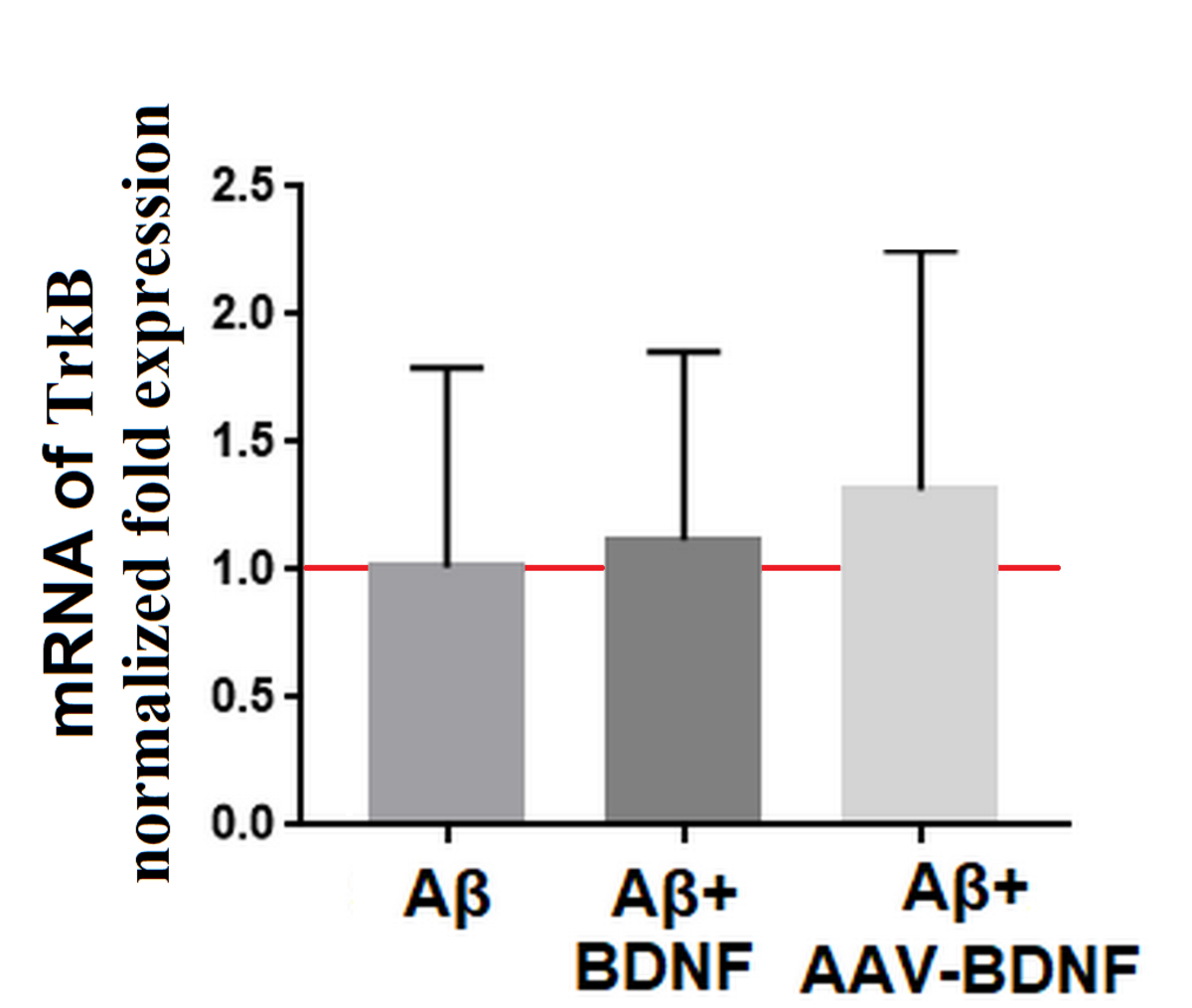

Supplement: FIGURE S2 — Features of TrkB-FL gene expression on DIV 21 under chronic exogenous administration of Aβ and BDNF. Data are normalized to the reference gene (Oaz1). The data represent the mean values ± SEMs from three independent experiments. We showed no significant alterations in TrkB-FL mRNA levels in primary hippocampal cultures on DIV21 under chronic Aβ application. [file Image_2.TIF]
